# Dietary Oyster Mushroom (*Pleurotus ostreatus*) Waste Inhibits Experimentally Induced *Eimeria tenella* Challenge in Japanese Quails Model

**DOI:** 10.3390/ani13213421

**Published:** 2023-11-04

**Authors:** Jamal Abdul Nasir, Naila Chand, Shabana Naz, Ibrahim A. Alhidary, Rifat U. Khan, Sajida Batool, Noha T. Zelai, Gianluca Pugliese, Vincenzo Tufarelli, Caterina Losacco

**Affiliations:** 1 Department of Poultry Science, Faculty of Animal Husbandry and Veterinary Sciences, The University of Agriculture, Peshawar 25130, Pakistan; jamalbatani@yahoo.com (J.A.N.); draleeze@yahoo.com (N.C.); 2 Department of Zoology, Government College University, Faisalabad 38000, Pakistan; 3 Department of Animal Production, College of Food and Agriculture, King Saud University, Riyadh 11362, Saudi Arabia; 4 College of Veterinary Sciences, Faculty of Animal Husbandry and Veterinary Sciences, The University of Agriculture, Peshawar 25130, Pakistan; 5 Department of Biological Sciences, Faculty of Science, King Abdulaziz University, Jeddah 21589, Saudi Arabia; 6 Section of Veterinary Science and Animal Production, Department of Precision and Regenerative Medicine and Jonian Area, University of Bari ‘Aldo Moro’, Valenzano, 70010 Bari, Italy; gianluca.pugliese1997.mv@gmail.com (G.P.); caterina.losacco@uniba.it (C.L.)

**Keywords:** mushroom waste, coccidiosis, Japanese quails, lesion score, intestinal histology

## Abstract

**Simple Summary:**

This study investigated the potential of repurposing discarded *Pleurotus ostreatus* stem waste as a natural dietary supplement to enhance growth performance and support intestinal health in Japanese quails, offering a natural solution to experimentally induced coccidiosis. The inclusion of 3% *Pleurotus ostreatus* in their diet proved highly effective in alleviating the compromised growth rate induced by coccidial oocysts. This dosage led to a notable reduction in the lesion scores in the cecum and a decrease in oocyst shedding. Additionally, it played a crucial role in restoring the cecal morphology of Japanese quails. These findings highlight the promising role of *Pleurotus ostreatus* stem waste as a valuable dietary component, potentially contributing to the overall well-being and performance of quails, particularly in the face of coccidial challenges. This natural remedy presents an eco-friendly and cost-effective approach to poultry farming, utilizing otherwise discarded materials for significant benefits in bird health and productivity.

**Abstract:**

The aim of this study was to investigate the potential of dietary 3% oyster mushroom (*Pleurotus ostreatus*) waste in enhancing the anticoccidial effects in broilers challenged with *Eimeria tenella* infection. The experiment involved a total of 600 Japanese quails, raised from one to thirty-five days of age, which were divided into four treatment groups. These included a negative control group that received a basal diet (BD) without any anticoccidial or antibiotic supplementation in the non-challenged birds (negative control, NC); a positive control (PC) group consisting of NC birds challenged with *E. tenella*; a group that received the BD with an anticoccidial drug (standard); and a group that received the BD supplemented with 3% waste from oyster mushrooms (3% *Pleurotus ostreatus*). The results showed that the feed intake, body weight gain, and feed efficiency were significantly lower in the PC (*p* < 0.05). However, the growth traits were similar in the standard and 3% *Pleurotus ostreatus*-treated groups. Similarly, there was no difference (*p* < 0.05) in the mortality rate, oocyst count in the feces, and lesion score between the standard and 3% *Pleurotus ostreatus* groups. Based on intestinal histology evaluation, the villi height and width were significantly higher in the standard and 3% *Pleurotus ostreatus*-treated groups compared to those of the PC (*p* < 0.01). In conclusion, it was found that 3% *Pleurotus ostreatus* effectively mitigated the low growth rate of Japanese quails induced by coccidial infection.

## 1. Introduction

Parasitic diseases affect living organisms worldwide. Various types of parasites threaten animals’ health in almost every country, and parasitic infections frequently hinder the growth of animals and plants [1,2]. The poultry industry is one of the vital component of global agriculture, providing a valuable source of animal protein for humans through consuming poultry meat [3,4]. Despite its rapid global expansion, the commercial poultry industry still faces numerous challenges, with coccidiosis being a significant obstacle to its advancement (Saeeda et al. 2023). Among the parasitic diseases, coccidiosis stands out as the most critical issue in poultry farming, caused by obligatory intestinal protozoans of the Eimeria species [5]. Coccidiosis typically results in intestinal damage, leading to poor feed efficiency and inflammation of both the small and large intestines and diarrhea. In severe cases, it can even lead to bird mortality, which is a well-documented fact [6]. The avian intestinal tract accommodates seven distinct species of avian coccidian, each exhibiting a particular site of preference [7]. Among these, *E. tenella* is the most prevalent and significant species in the poultry industry, which leads to substantial economic losses due to medication costs, a compromised FCR, and an increased bird mortality rate [8]. This species is hemorrhagic and primarily resides in the epithelial cells of the ceca. It causes the destruction of villi, resulting in weakness, diarrhea, and ultimately, the death of the chickens [9]. The traditional method of giving animals sub-therapeutic doses of antibiotics in food for safeguarding against coccidiosis and the overall enhancement of intestinal health is now facing scrutiny. With apprehensions regarding drug resistance and the recent ban on antibiotics as feed supplements in the European Union, there has been a discernible decline in animal health, leading to an increase in enteric diseases [10]. These factors have compelled researchers to seek out alternative strategies against infectious diseases [11].

Other studies have investigated natural alternatives to antibiotics, focusing on bioactive compounds derived from specific fungi, commonly referred to as medicinal mushrooms [12]. These fungi contain bioactive elements, like glucans, blazeispirol, and proteoglycans, known for their gastrointestinal immune-stimulating properties and potential to strengthen the immune system [13,14]. Furthermore, the inclusion of essential nutrients in these compounds may enhance the bacterial profiles, improving their digestibility in the gastrointestinal tract, and ultimately fostering the growth of broilers [15,16]. The surplus availability of *Pleurotus ostreatus* stem waste is attributed to the increasing cultivation of mushrooms. Despite them possessing medicinal and nutritional benefits, these stems are typically regarded as agricultural by-products. Furthermore, the increased popularity of mushrooms for human consumption has been associated with environmental pollution [17,18]. However, it is worth noting that the utilization of *Pleurotus ostreatus* stem waste in farm animal production is currently not widespread. Despite this, several studies have highlighted the positive effects of mushrooms on broilers’ performance, including improvements in immune response modulation, intestinal microbiota, enhanced antioxidant activity, the betterment of the lipid profile, and their influence on the intestinal morphology [19,20,21,22,23]. Considering the crucial role of the avian gastrointestinal tract in immune functions, this study endeavors to evaluate the feasibility of integrating mushroom (*Pleurotus ostreatus*) stem waste as a phytogenic feed supplement. This has the potential to serve as a substitute for antibiotics in broiler diets. To the best of our knowledge, there have been a limited number of studies conducted on evaluating the impact of mushroom waste on the performance and health of broilers.

Therefore, the objective of this study was to explore the potential of utilizing discarded *Pleurotus ostreatus* stem waste as a natural dietary supplement. Moreover, the goal was to sustain the growth performance and support the intestinal health of quails, serving as a natural remedy against experimentally induced coccidiosis.

## 2. Materials and Methods

### 2.1. Animals, Management and Treatments

This study involved a total of 600 Japanese quails, which were raised from one to forty-two days old. They were housed in 40 pens, with each pen accommodating 15 birds. Upon hatching, the birds were randomly allocated using a completely randomized block design into four treatment groups (150 birds/treatment), each with ten replicates. These treatments included a negative control group that received a basal diet (BD) without any anticoccidial challenged birds (NC); a positive control group (PC) consisting of NC birds challenged with *Eimeria tenella*; a group that received the BD with an anticoccidial drug (standard); and a group that received the BD supplemented with 3% of stem waste from oyster mushrooms (3% *Pleurotus ostreatus*). The anticoccidial used was Amprolium (Huvepharma Inc., Peachtree city, GA, USA), with an inclusion rate of 1.25 g/L. The stems of *P. ostreatus* were dried at 55 °C for 72 h, after which they were crushed and incorporated into the feed ration.

To avoid cross-contamination between the pens subjected to the challenge and those without, distinct plastic boots were assigned for each group. One set of boots was exclusively used for the challenged pens, while the other set was worn in the non-challenged pens. Furthermore, trays containing calcium oxide were placed at the entrance of the pens housing the non-challenged birds, acting as a footbath to provide an additional barrier against potential contamination.

The average temperature and humidity of the facility was 27.75 ± 2.4°C and 65%, respectively. Water and feed were made available without restriction. The feeding regimen was divided into two phases: the starter phase (from day 1 to day 21) and the finisher phase (from day 22 to day 35). The rations provided were designed to be maintain isoenergetic and consistent levels of amino acids. They were prepared using a combination of corn and soybean meal, as detailed in Table 1. On the 14th day of the experiment, each bird received an oral inoculation of 0.5 mL solution containing 2.5 × 10^5^ sporulated oocysts of *E. tenella*. To ensure unbiased results, the birds not subjected to the challenge were similarly inoculated with a saline solution, exposing them to the same handling stress.

### 2.2. Performance Traits

The performance variables assessed included body weight gain, average feed intake, feed conversion ratio (FCR), and mortality over the 35-day period. At the study’s conclusion, three birds/replicates were left to fast for 12 h. The quails were slaughtered with a sharp knife, and the skin was eviscerated. The heads, feet, and visceral organs were removed. The dressing percentage was calculated as (eviscerated weight/live weight) × 100.

### 2.3. Lesion Score and Oocyst Count

The lesion score was determined on 6th day post infection (DPI). Two quails per replicate were chosen for lesion scoring. The assessment was conducted following the method outlined by Johnson and Ried [24].This approach involves grading lesions on a scale from 0 to 4 based on their severity. A sample exhibiting no lesions received a grade of 0, while those with slight lesions were designated as grade 1. Samples displaying mild lesions were assigned grade 2, and those with moderate lesions were given grade 3. Samples exhibiting severe lesions were recorded as grade 4.

Fecal samples were collected from the birds on 5, 7, and 9 DPI. To collect feces for oocyst per gram (OPG), fecal material from various areas of the pen to ensure a comprehensive representation were collected. The litter was carefully scooped, making sure to avoid any contamination from non-fecal matter. The collected samples were transferred to a clean, labeled container. The samples were thoroughly homogenized. From this homogenized mixture, a subsample was taken for further processing and analysis to determine the OPG. This collection process took place in the evening, and the samples were promptly chilled and stored overnight. The following day, oocysts in the collected samples were quantified using the MacMaster technique [2].

### 2.4. Intestinal Histomorphology

Morphological examination was conducted on day 35 utilizing light microscopy. For each treatment, six birds were selected and sacrificed. From each bird, a 3 cm segment of the cecum was collected. These segments were rinsed in physiological solution, and then fixed in 10% formaldehyde for 24 h and subsequently stored in 70% alcohol. The samples underwent a dehydration process in alcohol, progressing from lower to higher concentrations (70–100%). They were then made to be translucent in xylene and embedded in histological paraffin. The slides were 5-micrometer thick sections of each segment, which were then stained using hematoxylin & eosin (HE). Using an optical microscope (Nikon, Tokyo, Japan), a total of 10 views were captured per sample for measurements of the villi height, width, and crypt depth. These parameters were employed to calculate the villus height to crypt depth ratio.

### 2.5. Statistical Analysis

Data analysis was conducted utilizing SAS statistical software (version 2012), with a significance level of 5%. Initially, normality tests for the residuals and assessments of homogeneity of variances were executed. Subsequently, the data underwent analysis of variance using the GLM procedure. The pens were considered as the experimental unit. In instances where a significant effect was observed, the means were compared using Tukey’s test (*p* < 0.05).

## 3. Results

The impact of the stem waste powder from oyster mushrooms (*Pleurotus ostreatus*) on the feed intake of quails challenged with *Eimeria tenella* is detailed in Table 2. While no significant effect on feed intake was observed on week 2, notably higher feed intake was recorded in the NC (non-challenged) and standard groups during week 3 and in the starting phase. In weeks 4, 5, and 6, a significantly higher feed intake was noted in the NC, standard, and 3% *Pleurotus ostreatus* groups (*p* < 0.05). Ultimately, at the conclusion of the study (over the entire period), a significantly higher feed intake was observed in the NC and standard groups compared to that of the PC (challenged) group (*p* < 0.05).

The impact of the stem waste powder from oyster mushrooms (*Pleurotus ostreatus*) on the weight gain of quails challenged with *Eimeria tenella* is presented in Table 3. Similar to feed intake, weight gain showed no significant differences between the groups on the second week. However, it was significantly higher (*p* < 0.05) in the NC (non-challenged), standard, and 3% *Pleurotus ostreatus* groups. Overall, at the conclusion of the starting phase, a significantly higher weight gain was observed in the NC and standard groups compared to that in the PC (challenged) group (*p* < 0.05). On weeks 4, 5, and 6, a significantly higher weight gain was recorded in the NC, standard, and 3% *Pleurotus ostreatus* groups (*p* < 0.05).

During the finish phase, a higher weight gain was noted only in the NC and 3% *Pleurotus ostreatus* groups. Overall, a significantly higher weight gain was observed in the NC group compared to those of the rest of the groups (*p* < 0.05); however, the weight gain was also significantly higher in the standard and 3% *Pleurotus ostreatus* groups (*p* < 0.05).

The influence of the stem waste powder from oyster mushrooms (*Pleurotus ostreatus*) on the feed conversion ratio (FCR) of quails challenged with *Eimeria tenella* is outlined in Table 4. On a weekly basis, as well as during the starting and finish phases and the overall period, the FCR was significantly higher (*p* < 0.05) in the PC (challenged) group compared to that of the other groups.

The impact of oyster mushroom stem waste powder on the mortality and dressing percentage of quails challenged with *Eimeria tenella* is presented in Table 5. The mortality rate was notably higher in the positive control (PC) group compared to those of all the other groups, showing statistical significance (*p* = 0.03).

Likewise, the dressing percentage was significantly lower in the PC group (*p* < 0.01). The standard and the 3% *Pleurotus ostreatus* groups exhibited similar dressing percentages and mortality rates.

The effect of oyster mushroom stem waste powder on the oocyst count per gram of feces in quails challenged with *Eimeria tenella* is presented in Table 6. The results indicate that the oocyst count per gram (OPG) was significantly higher in the positive control (PC) group at 5, 7, and 9 days post infection (DPI) (*p* < 0.01). However, in the birds fed 3% *Pleurotus ostreatus*, the OPG significantly decreased at 10 and 20 DPI (*p* < 0.01), showing similarity to the standard group.

The effect of oyster mushroom stem waste powder on the lesion score of the ceca in the quails challenged with *Eimeria tenella* is outlined in Table 7. The positive control (PC) group displayed a higher score. However, the lesion scores were comparable between the standard and 3% *Pleurotus ostreatus*-treated birds.

**Figure 1:**
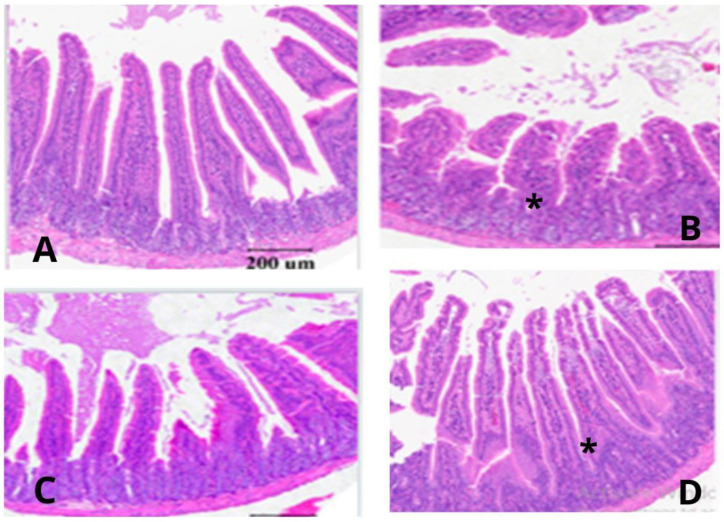
(**A**) Photomicrograph of cecal villi in unchallenged and untreated Japanese quails (NC), displaying nearly normal villi. (**B**) Photomicrograph of cecal villi in quails challenged with *E. tenella* and treated with 3% *Pleurotus ostreatus* stem waste, revealing no significant lesions related to coccidiosis, but with slight infiltration of reactive cells in the lamina propria. A few merozoites/schizonts were observed (*); (**C**) Photomicrograph of cecal villi in quails challenged with *E. tenella* and treated with Amprolium (standard), showing villi similar to those in the unchallenged group (NC). (**D**) Photomicrograph of cecal villi in quails challenged with *E. tenella* (PC), displaying the erosion and desquamation of crypt epithelia, along with infiltration of reactive cells in the lamina propria. Merozoites/schizonts were also observed in the crypt of the villi. merozoites/schizont in the cecal crypt (*). Bar: 200 µm.

The oyster (*Pleurotus ostreatus*) mushroom stem waste effects on the cecal villi height, crypt depth, villus-height-to-crypt-depth ratio, and width of Japanese quails challenged with *Eimeria tenella* are detailed in Table 8 and Figure 1. The groups fed a normal diet (NC) and 3% *Pleurotus ostreatus* showed a significantly higher villus height and width (*p* < 0.01). Conversely, the 3% *Pleurotus ostreatus* group exhibited a significantly lower crypt depth compared to that of the positive control (PC) group (*p* < 0.01). Likewise, the VH:CD ratio was significantly higher in the 3% *Pleurotus ostreatus* group compared to that of the PC group (*p* < 0.01).

## 4. Discussion

For many decades, the conventional methods of controlling coccidiosis have primarily depended on the use of anticoccidial chemical compounds and live vaccines. However, the emergence of drug-resistant strains of Eimeria and growing apprehension regarding the presence of drug residues in animal products prompted researchers to explore alternatives. This has led to the investigation of plant extracts, which contain numerous compounds that are both safe and cost-effective, offering a potentially viable solution [25]. In the present study, it was observed that the growth performance of the positive control group (PC) was adversely affected when compared to that of the other treatment groups. The observed decline in growth performance could be attributed to the detrimental impact of coccidial parasites on the intestines. This may result in a diminished appetite and the impaired absorption of nutrients. Interestingly, the growth performance showed significant improvement in the group fed 3% *Pleurotus ostreatus*. Ongoing debates surround the performance and physiological responses of broilers when fed different medicinal mushrooms, as indicated by the findings from prior studies. These discussions arise from various variables, including the mushroom species, administered doses, application methods (whether non-fermented or fermented and in conjunction with other beneficial organisms), the specific parts of mushrooms used (such as fruiting bodies or stem bases), and the duration of the treatment. However, there is a general consensus among many scientists that mushrooms may indeed have a positive influence on enhancing the performance and overall health status of broiler chickens [21,25,26].

In the current study, incorporating 3%*Pleurotus ostreatus* into the diet led to a noteworthy reduction in the lesion scores, mortality rate, and oocyst per gram (OPG). Evaluating lesion grading is pivotal in gauging the intensity of coccidiosis infection. Birds subjected to the challenge with lowest lesion scores in their ceca showed minor damage, indicating the higher probability of successful recovery [27]. During necropsy, it was observed that the hemorrhagic lesions improved in all the treated groups, particularly in the 3% P. ostreatus group. The improvement of lesions is likely due to a reduction in the number of Eimeria sporozoites causing the damage. Conversely, the untreated group consistently exhibited numerous petechial hemorrhages throughout the study, indicating the detrimental impact of coccidial parasites. The observed lesions during necropsy confirmed the presence of *Eimeria tenella* infection in the broilers. These findings are similar to the conclusion of Ademola et al. [25] that an aqueous extract of *P. ostreatus* reduced the lesion score on day 35 of the coccidial challenge in the broiler chickens. These findings indicate that incorporating 3% *Pleurotus ostreatus* into the chickens’ diet may help mitigate the challenge presented by *E. tenella*, potentially by bolstering their immune functions. Mushrooms contain various compounds, such as glycosides, alkaloids, polysaccharides, organic acids, and volatile oils, that have been shown to stimulate the immune system [28]. Other research studies have demonstrated that the presence of poly- and oligosaccharides in mushrooms can affect both adaptive and innate immunity, including the humoral and cellular responses [29]. Additionally, mushrooms are a rich source of selenium, a trace element that enhances the antioxidant potential [30], consequently bolstering the immunity of animals that incorporate it into their diet.

The present morphometric analysis of cecum revealed reduced villi height, width and higher crypt depth in the birds challenged with *E. tenella*, suggesting they were subjected to challenging conditions, likely due to the presence of undesirable microorganisms. This led to heightened cell proliferation as a response. Conversely, a lower crypt depth and increased villi height and width were observed in the birds treated with 3% *Pleurotus ostreatus*, which may indicate improved intestinal health. This suggests that cell proliferation continued to occur, potentially to compensate for cell loss and maintain the integrity of the villous apical region [22]. Coccidiosis commonly leads to a reduction in the growth rate due to a reduced feed intake and body weight. This is attributed to Eimeria’s ability to cause damage to the anatomical structures of the intestines, resulting in the decreased absorption of nutrients [10]. However, in our present study, we observed a reversal in the necrotic tissue of the ceca when 3% *Pleurotus ostreatus* was included in the diet during the coccidial challenge. Increased villus dimensions are associated with the enhanced absorption of nutrients in the gastrointestinal tract [10]. In the 3% *Pleurotus ostreatus* group, the ceca displayed an almost normal structure, similar to that observed in the normal control (NC) group. The histopathological examination of the ceca in the NC group did not reveal any significant lesions associated with coccidiosis, but there was a slight infiltration of reactive cells in the lamina propria. Hence, employing 3% *Pleurotus ostreatus* appears to be a promising alternative for treating coccidiosis in poultry production. It is recommended to conduct additional studies to elucidate the mechanism of action of *P. ostreatus*. Utilizing bioactivity-guided fractionation could provide a more in-depth understanding of the specific active component(s) responsible for its anticoccidial and immune-enhancing properties.

## 5. Conclusions

In the current study, it was observed that the inclusion of 3% *Pleurotus ostreatus* can effectively mitigate the low growth rate of quails caused by Japanese quails ingesting coccidial oocysts. Additionally, this dose demonstrated a reduction in lesion scores in the cecum and a decrease in oocyst shedding. Furthermore, 3% *Pleurotus ostreatus* also contributed to the restoration of villi dimensions in the ceca of Japanese quails. Future studies should focus on elucidating the regulatory mechanisms that govern gene expression, including epigenetic modifications and transcription factor interactions. Investigating the impact of environmental factors, such as stressors or dietary components, on gene expression will provide a comprehensive understanding. Additionally, exploring gene expression in specific tissues or cell types can offer targeted insights into biological processes.

## Figures and Tables

**Table 1:** Ingredients and chemical composition of basal diet.

Ingredients	Starter Phase (1–21 Days)	Finisher Phase (22–35 Days)
Yellow corn	54.00	59.75
Soybean meal (48% CP)	36.50	26.00
Corn gluten meal	2.50	7.25
Corn oil	2.50	2.80
Dicalcium phosphate	2.35	2.15
Limestone	0.85	0.70
NaCl	0.20	0.20
Vitamin and minerals premix ^1^	0.50	0.50
DL-Methionine	0.25	0.15
L-Lysine HCl	0.21	0.35
L-Threonine	0.12	0.11
Choline chloride	0.05	0.05
**Chemical composition**		
ME, kcal/kg	3000	3150
Crude protein, %	22.5	21.30
Methionine, %	0.55	0.44
Lysine, %	1.42	1.23
Sulfur amino acids, %	0.96	0.80
Threonine, %	0.95	0.85
Calcium, %	1.05	0.90
Available phosphorus, %	0.50	0.45

^1^ Provided per kg of diet: vitamin A, 10,000 IU; vitamin E, 30 IU; vitamin D, 30,000 IU; thiamine, 2.5 mg; vitamin K3, 2.5 mg; pyridoxine, 5 mg; riboflavin, 5.5 mg; folic acid, 0.7 mg; vitamin B12, 0.5 mg; pantothenic acid, 15 mg; nicotinic acid, 50 mg; biotin, 0.2 mg; copper, 12 mg; manganese, 100 mg; iron, 95 mg; selenium, 0.5 mg; iodine, 0.5 mg; zinc, 100 mg.

**Table 2:** Effect of stem waste powder of oyster (*Pleurotus ostreatus*) mushroom on feed intake (g/bird) of quails challenged with *Eimeria tenella*.

Phases	Negative Control	Positive Control	Standard	3% *Pleurotus ostreatus*	*p*-Value
Week-2	50.0 ± 1.52	45.3 ± 1.45	48.0 ± 1.52	43.3 ± 2.33	0.1077
Week-3	97.0 ± 1.76 ^a^	85.3 ± 3.71 ^b^	91.3 ± 2.60 ^ab^	90.0 ± 0.57 ^ab^	0.0293
Starter phase	147.0 ± 1.45 ^a^	130.7 ± 2.60 ^b^	139.3 ± 4.09 ^ab^	133.3 ± 2.90 ^b^	0.0211
Week-4	122.0 ± 2.08 ^a^	101.3 ± 2.02 ^b^	123.0 ± 5.23 ^a^	117.3 ± 2.33 ^a^	0.0011
Week-5	143.3 ± 4.25 ^a^	106.3 ± 4.25 ^b^	136.0 ± 6.55 ^a^	135.0 ± 3.46 ^a^	0.0014
Week-6	159.0 ± 3.05 ^a^	113.7 ± 3.84 ^b^	157.7 ± 1.33 ^a^	153.7 ± 0.88 ^ab^	<0.0001
Finisher Phase	424.3 ± 2.02 ^a^	321.3 ± 5.23 ^c^	416.0 ± 8.00 ^ab^	406.0 ± 4.04 ^b^	<0.0001
Overall	571.3 ± 5.91 ^a^	452.0 ± 6.08 ^c^	556.0 ± 5.48 ^ab^	539.3 ± 6.43 ^b^	<0.0001

Means in the same row with different superscripts are significantly different at *p* < 0.05. Negative control: basal diet without any anticoccidial-challenged birds. Positive control: negative control birds challenged with *E. tenella*. Standard: basal diet with an anticoccidial drug. The 3% *Pleurotus ostreatus* group: basal diet supplemented with 3% of stem waste from oyster mushrooms.

**Table 3:** Effect of stem waste powder of oyster (*Pleurotus ostreatus*) mushroom on weight gain (g/bird) of quails challenged with *Eimeria tenella*.

Phases	Negative Control	Positive Control	Standard	3% *Pleurotus ostreatus*	*p*-Value
Week-2	28.0 ± 2.08	25.3 ± 1.15	27.3 ± 2.84	28.0 ± 1.73	0.7030
Week-3	41.0 ± 2.51 ^a^	27.3 ± 0.88 ^b^	38.3 ± 2.02 ^a^	35.7 ± 1.76 ^a^	0.0047
Starter phase	69.0 ± 0.57 ^a^	52.3 ± 2.02 ^c^	65.7 ± 1.85 ^ab^	63.7 ± 0.33 ^b^	0.0002
Week-4	42.3 ± 2.66 ^a^	25.7 ± 2.02 ^b^	38.3 ± 1.45 ^a^	36.3 ± 2.90 ^a^	0.0055
Week-5	44.3 ± 2.96 ^a^	24.3 ± 0.33 ^b^	39.7 ± 1.33 ^a^	42.0 ± 1.52 ^a^	0.0002
Week-6	38.7 ± 2.02 ^a^	25.3 ± 1.45 ^b^	35.7 ± 1.45 ^a^	38.7 ± 1.76 ^a^	0.0015
Finisher Phase	125.7 ± 1.76 ^a^	75.3 ± 1.45 ^c^	113.0 ± 2.33 ^b^	117.0 ± 5.1 ^ab^	<0.0001
Overall	194.7 ± 1.20 ^a^	127.7 ± 3.18	179.3 ± 0.66 ^b^	180.7 ± 5.04 ^b^	<0.0001

Means in the same row with different superscripts are significantly different at *p* < 0.05. Negative control: basal diet without any anticoccidial-challenged birds. Positive control: negative control birds challenged with *E. tenella*. Standard: basal diet with an anticoccidial drug. The 3% *Pleurotus ostreatus* group: basal diet supplemented with 3% of stem waste from oyster mushrooms.

**Table 4:** Effect of stem waste powder of oyster (*Pleurotus ostreatus*) mushroom on feed conversion ratio of quails challenged with *Eimeria tenella*.

Phases	Negative Control	Positive Control	Standard	3% *Pleurotus ostreatus*	*p*-Value
Week-2	1.8 ± 0.12	1.8 ± 1.45	1.8 ± 1.52	1.5 ± 0.07	0.5160
Week-3	2.4 ± 0.08 ^b^	3.1 ± 0.17 ^a^	2.4 ± 0.11 ^a^	2.5 ± 0.13 ^b^	0.0105
Starter phase	2.1 ± 0.03 ^b^	2.5 ± 0.12 ^a^	2.1 ± 0.12 ^b^	2.1 ± 0.05 ^b^	0.0438
Week-4	2.9 ± 0.14 ^b^	3.9 ± 0.28 ^a^	3.2 ± 0.21 ^b^	3.3 ± 0.20 ^b^	0.0381
Week-5	3.2 ± 0.24 ^b^	4.4 ± 0.16 ^a^	3.4 ± 0.05 ^b^	3.2 ± 0.18 ^b^	0.0049
Week-6	4.1 ± 0.15	4.4 ± 0.10	4.4 ± 0.13	3.9 ± 0.14	0.0873
Finisher phase	3.4 ± 0.04 ^b^	4.3 ± 0.14 ^a^	3.7 ± 0.11 ^b^	3.5 ± 0.14 ^b^	0.0030
Overall	2.9 ± 0.02 ^b^	3.5 ± 0.12 ^a^	3.1 ± 0.03 ^b^	2.9 ± 0.06 ^b^	0.0018

Means in the same row with different superscripts are significantly different at *p* < 0.05. Negative control: basal diet without any anticoccidial-challenged birds. Positive control: negative control birds challenged with *E. tenella*. Standard: basal diet with an anticoccidial drug. The 3% *Pleurotus ostreatus* group: basal diet supplemented with 3% of stem waste from oyster mushrooms.

**Table 5:** Effect of stem waste powder of oyster (*Pleurotus ostreatus*) mushroom on mortality and dressing percentage of quails challenged with *Eimeria tenella*.

Groups	Mortality Rate, %	Dressing Yield, %
Negative Control	5.0 ± 0.00 ^b^	70.0 ± 0.96 ^a^
Positive Control	20.0 ± 5.77 ^a^	61.2 ± 1.09 ^c^
Standard	8.3 ± 1.66 ^b^	66.7 ± 1.04 ^b^
3% *Pleurotus ostreatus*	6.7 ± 0.33 ^b^	67.0 ± 0.86 ^b^
*p*-value	0.0345	0.0011

Means in the same row with different superscripts are significantly different at *p* < 0.05. Negative control: basal diet without any anticoccidial-challenged birds. Positive control: negative control birds challenged with *E. tenella*. Standard: basal diet with an anticoccidial drug. The 3% *Pleurotus ostreatus* group: basal diet supplemented with 3% of stem waste from oyster mushrooms.

**Table 6:** Effect of stem waste powder of oyster (*Pleurotus ostreatus*) mushroom on oocyst count per gram of feces in Japanese quails challenged with *Eimeria tenella*.

Groups	5 DPI	7 DPI	9 DPI
Negative Control	0 ± 0.0 ^c^	0 ± 0.0 ^c^	0 ± 0.0 ^b^
Positive Control	243.0 ± 48.88 ^a^	567.7 ± 76.52 ^a^	292.7 ± 82.64 ^a^
Standard	105.0 ± 4.04 ^b^	177.0 ± 32.62 ^b^	104.7 ± 25.94 ^b^
3% *Pleurotus ostreatus*	150.0 ± 40.52 ^ab^	220.7 ± 1.66 ^b^	137.3 ± 0.98 ^b^
*p*-value	0.0042	0.0003	0.0147

Means in the same row with different superscripts are significantly different at *p* < 0.05. DPI: days post inoculation. Negative control: basal diet without any anticoccidial-challenged birds. Positive control: negative control birds challenged with *E. tenella*. Standard: basal diet with an anticoccidial drug. The 3% *Pleurotus ostreatus* group: basal diet supplemented with 3% of stem waste from oyster mushrooms.

**Table 7:** Effect of stem waste powder of oyster (*Pleurotus ostreatus*) mushroom on lesion score of ceca of quails challenged with *Eimeria tenella*.

Groups	Hemorrhages	Congestion
Negative Control	0 ± 0.0	0 ± 0.0
Positive Control	4 ± 0.1	4 ± 0.1
Standard	1 ± 0.2	1 ± 0.1
3% *Pleurotus ostreatus*	1 ± 0.1	1 ± 0.1

Negative control: basal diet without any anticoccidial-challenged birds; Positive control: negative control birds challenged with *E. tenella*. Standard: basal diet with an anticoccidial drug. The 3% *Pleurotus ostreatus* group: basal diet supplemented with 3% of stem waste from oyster mushrooms.

**Table 8:** Effect of stem waste of oyster (*Pleurotus ostreatus*) mushroom on cecal histomorphology of Japanese quails challenged with *Eimeria tenella*.

Groups	Villus Height (µm)	Crypt Depth (µm)	VH:CD	Villus Width (µm)
Negative Control	841 ± 7.12 ^a^	159.1 ± 1.32 ^b^	5.29 ± 0.96 ^a^	180.3 ± 4.81 ^a^
Positive Control	828 ± 8.32 ^c^	116.6 ± 2.77 ^a^	7.10 ± 0.65 ^c^	110.5 ± 3.22 ^c^
Standard	833 ± 6.67 ^b^	151.5 ± 2.54 ^b^	5.49 ± 0.49 ^b^	165.4 ± 3.11 ^ab^
3% *Pleurotus ostreatus*	837 ± 8.66 ^ab^	142.4 ± 2.55 ^b^	5.88 ± 0.44 ^b^	157.9- ± 5.41 ^b^
*p*-value	0.0002	0.0217	0.0396	0.0029

Means in the same row with different superscripts are significantly different at *p* < 0.05. VH:CD, Villus-height-to-crypt-depth ratio. Negative control: basal diet without any anticoccidial-challenged birds. Positive control: negative control birds challenged with *E. tenella*. Standard: basal diet with an anticoccidial drug. The 3% *Pleurotus ostreatus* group: basal diet supplemented with 3% of stem waste from oyster mushrooms.

## Data Availability

Not applicable.
